# Concerted conservation actions to support chimpanzee cultures

**DOI:** 10.1098/rstb.2024.0143

**Published:** 2025-05-01

**Authors:** Erin G. Wessling, Andrew Whiten, Serge K. Soiret, Katy Scholfield, Liran Samuni, Christian Rutz, Ian Redmond, Lilian Pintea, Annette Lanjouw, Kathelijne Koops, Serge A. Kamgang, Ammie K. Kalan, Rachel Ashegbofe Ikemeh, Tatyana Humle, Catherine Hobaiter, Heidrun Frisch-Nwakanma, Elodie Freymann, Osiris Doumbe, Philippa Brakes, Ekwoge Abwe, Crickette Sanz

**Affiliations:** ^1^ School of Psychology and Neuroscience, University of St Andrews, St Andrews, UK; ^2^ Cognitive Ethology Lab, Deutsches Primatenzentrum GmbH - Leibniz-Institut für Primatenforschung, Göttingen, Niedersachsen, Germany; ^3^ Senckenberg Museum für Naturkunde Görlitz, 02826 Görlitz, Germany; ^4^ Department of Human Evolutionary Biology, Harvard University, Cambridge, MA 02138, USA; ^5^ Cooperative Evolution Lab, Deutsches Primatenzentrum GmbH - Leibniz-Institut für Primatenforschung, Gottingen, Niedersachsen 37077, Germany; ^6^ Centre de Recherche en Ecologie (CRE) / Nangui Abrogoua University, 02 B.P. 801 Abidjan 02, Côte d'Ivoire; ^7^ Arcus Foundation, New York, NY 10001, USA; ^8^ Centre for Biological Diversity, School of Biology, University of St Andrews, St Andrews KY16 9JP, UK; ^9^ Ecoflix Foundation, Los Angeles, CA 91302, USA; ^10^ Ape Alliance, Stroud, UK; ^11^ Conservation Science Department, The Jane Goodall Institute, Washington, DC 20036, USA; ^12^ Ape Behaviour & Ecology Group, Department of Evolutionary Anthropology, University of Zurich, 8057 Zurich, Switzerland; ^13^ BSB Yamoussa, Deutsche Gesellschaft für Internationale Zusammenarbeit, Garoua-Plateau (Ecole de Faune), Cameroon; ^14^ Biodiversity-Environment & Sustainable Development, Garoua, Cameroon; ^15^ GAB Lab, Department of Anthropolgy, University of Victoria, Victoria, British Columbia, Canada V8W 2Y2; ^16^ SW/Niger Delta Forest Project, Abuja, Nigeria; ^17^ Re:wild, Austin, TX 78767, USA; ^18^ Secretariat Convention on the Conservation of Migratory Species of Wild Animals, 53113 Bonn, Germany; ^19^ School of Anthropology and Museum Ethnography, University of Oxford, Oxford OX2 6PE, UK; ^20^ Sekakoh Organization, Mankon, Cameroon; ^21^ Centre for Ecology and Conservation, University of Exeter, Penryn, Cornwall TR10 9FE, UK; ^22^ Cetacean Ecology Research Group, Massey University School of Natural Sciences, Albany, Auckland 0745, New Zealand; ^23^ Whale and Dolphin Conservation, Chippenham, Wiltshire SN15 1LJ, UK; ^24^ San Diego Zoo Wildlife Alliance, San Diego, CA 92027, USA; ^25^ Cameroon Biodiversity Association, Douala, Cameroon; ^26^ Department of Anthropology, Washington University in Saint Louis, St Louis, MO 63130, USA; ^27^ Congo Program, Wildlife Conservation Society, Brazzaville, Congo

**Keywords:** behavioural diversity, conservation policy, multi-scale conservation, social learning, behavioural monitoring, nut-cracking

## Abstract

Chimpanzees were among the first animals recognized to have culture, and our understanding of the breadth of their cultural repertoire has grown significantly since the 1960s. Throughout their range, chimpanzee populations have come under increasing pressure, with their endangered status necessitating immediate and long-term conservation interventions. Recognizing the importance of diverse behavioural repertoires for chimpanzees’ survival, there has been a recent focus of conservation efforts on preserving their culturally transmitted behaviours and the environments in which they are exhibited. This article evaluates the practicality of developing conservation measures focused on chimpanzee culture. We highlight innovative conservation strategies aimed at integrating chimpanzee cultural behaviours into conservation policies. We review synergistic conservation initiatives led by the International Union for Conservation of Nature, the UN Convention on the Conservation of Migratory Species of Wild Animals and other international and local groups that share the goal of preserving chimpanzee populations and their cultural diversity. We underline how successful conservation implementation requires engagement and collaboration with a diverse group of interested or affected people. Finally, we provide recommendations aimed at guiding future efforts to incorporate animal cultures into conservation strategies.

This article is part of the theme issue ‘Animal culture: conservation in a changing world’.

## 1. Introduction

### (a) Evidence for animal culture and the distinctive scale of chimpanzee culture research

Culture was once considered a Rubicon of human uniqueness, but over the past 60 years, a large body of research has demonstrated the existence of social learning and culture across vertebrate taxa [1]. Definitions of culture vary across academic disciplines; within evolutionary biology, culture has been defined as ‘those group-typical behaviour patterns shared by members of a community that rely on socially learned and transmitted information’ [2, p. 11]. Culture in this sense permeates numerous domains of animal behaviour, shaping behaviour not only during early development but also in later life (e.g. [3]) . Cultural processes can shape behavioural diversity and adaptability, potentially affecting survival [4,5]. Yet, animal culture has also been argued to possess ‘intrinsic value’ that is worth protecting in its own right [6,7]. These perspectives are not mutually exclusive, and when employed strategically, can be complementary.

Chimpanzees (*Pan troglodytes*) have been particularly instrumental in advancing our understanding of non-human culture. Studied in over 200 localities across sub-Saharan Africa [8,9], covering the species’ extensive geographical and ecological range, chimpanzees have evidenced distinctively rich and varied repertoires of traditions [9–11]. This cumulative knowledge has yielded formal frameworks for delineating and assessing cultural uniqueness [12] and confirmed the existence of broad geographical differences in the behavioural repertoires of wild chimpanzee communities. To date, more than 40 behavioural tool-use variants with a putative cultural component have been identified [9,10,12], and the list of non-foraging cultural candidates continues to grow (e.g. [13–15]).

Chimpanzee populations across their range exhibit unique arrays of traditions that are not readily attributed to environmental or genetic factors^1^ [12,17]. Such cultural differences are supported by studies on neighbouring communities in the wild (e.g. [15,18–20]) and in captive settings (e.g. [21,22]), where potential genetic and environmental influences can be more easily controlled. Researchers have documented multiple avenues of invention and transmission (e.g. spontaneous innovation [23]; inter-group horizontal transmission [24] and skill scaffolding [25]), subsequently replicated in experimental settings (e.g. [26]). Collectively, these powerfully convergent lines of evidence establish much of the observed chimpanzee behavioural diversity shared within communities as evidence of cultural richness.

### (b) The dire conservation status of chimpanzees and current conservation approaches

Tragically, the total estimated chimpanzee population in Africa has shrunk from 1−2 million individuals historically to fewer than 460 000 currently [27] and is predicted to decline by more than 50% over the next 75 years, with chimpanzees already extirpated from at least three countries [28,29]. The main threats to chimpanzees include disease, the pet trade, poaching and habitat loss [30]. In recent decades, industrial plantations and subsistence agriculture have increasingly encroached into ape habitats [27,30]. Extractive industries such as mining and logging and infrastructure developments (e.g. road construction) further threaten chimpanzees [27,30]. Chimpanzee ranges have reduced by as much as 70% in certain countries (e.g. Côte d’Ivoire [28,31]), and global issues like climate change are anticipated to exacerbate current pressures [29]. Only a few wild chimpanzee populations are under any kind of formal state protection; in the case of western chimpanzees, for example, national protected areas cover only 17% of their range [32].

**Figure 1.**
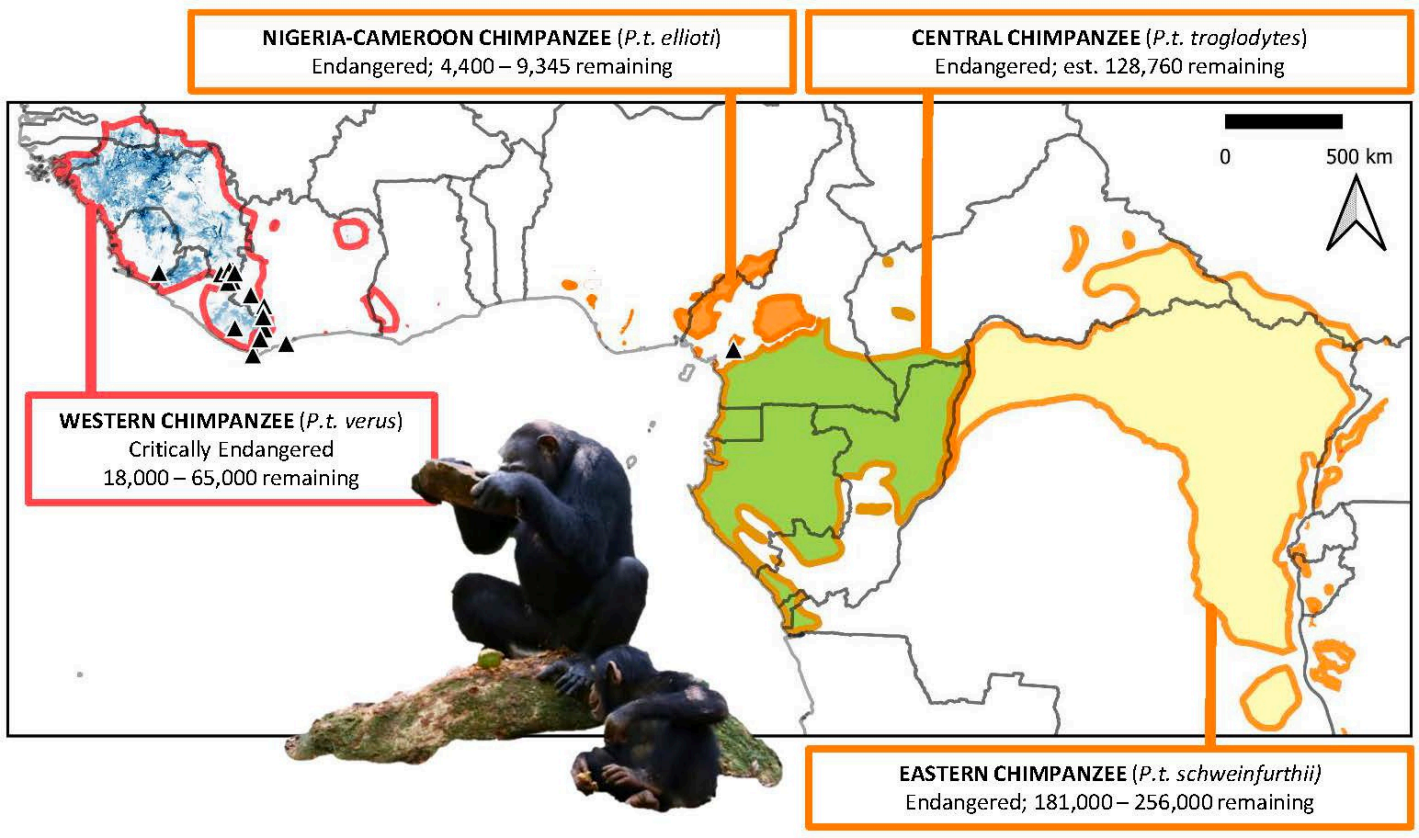
Distribution and status of the four subspecies of chimpanzee (source [27]), including the relative modelled density of western chimpanzees across their distribution (*P. t. verus*; visible scale in blue: 0.1–6.3 km^2^, adapted from [33]). Solid black triangles indicate reported nut-cracking locations (source [10]). Subspecies are outlined in colours corresponding to their *IUCN Red List* status, with red indicating ‘Critically Endangered’ and orange indicating ‘Endangered’ [30]. Photo inset: © Liran Samuni/Taï Chimpanzee Project.

Three chimpanzee subspecies (*Pan troglodytes ellioti*, *Pan troglodytes troglodytes* and *Pan troglodytes schweinfurthii*) are now classified as ‘Endangered’, while the western chimpanzee (*Pan troglodytes verus*) is listed as ‘Critically Endangered’ by the International Union for Conservation of Nature (IUCN)^2^ [30; figure 1]. The western subspecies experiences some of the most significant declines of the chimpanzee subspecies [31], as West Africa simultaneously undergoes significant land-use changes and human population growth [34]. Although this region has also historically received far less conservation funding and attention than other parts of Africa [28,35,36], the behavioural repertoire of this subspecies is among the most intensively studied in the wild (e.g. [9]). In addition, the subspecies comprises the greatest percentage of captive individuals [37]. Therefore, the ape subspecies whose cultural behaviour is among the best studied is also at the greatest risk of extinction.

**Figure 2.**
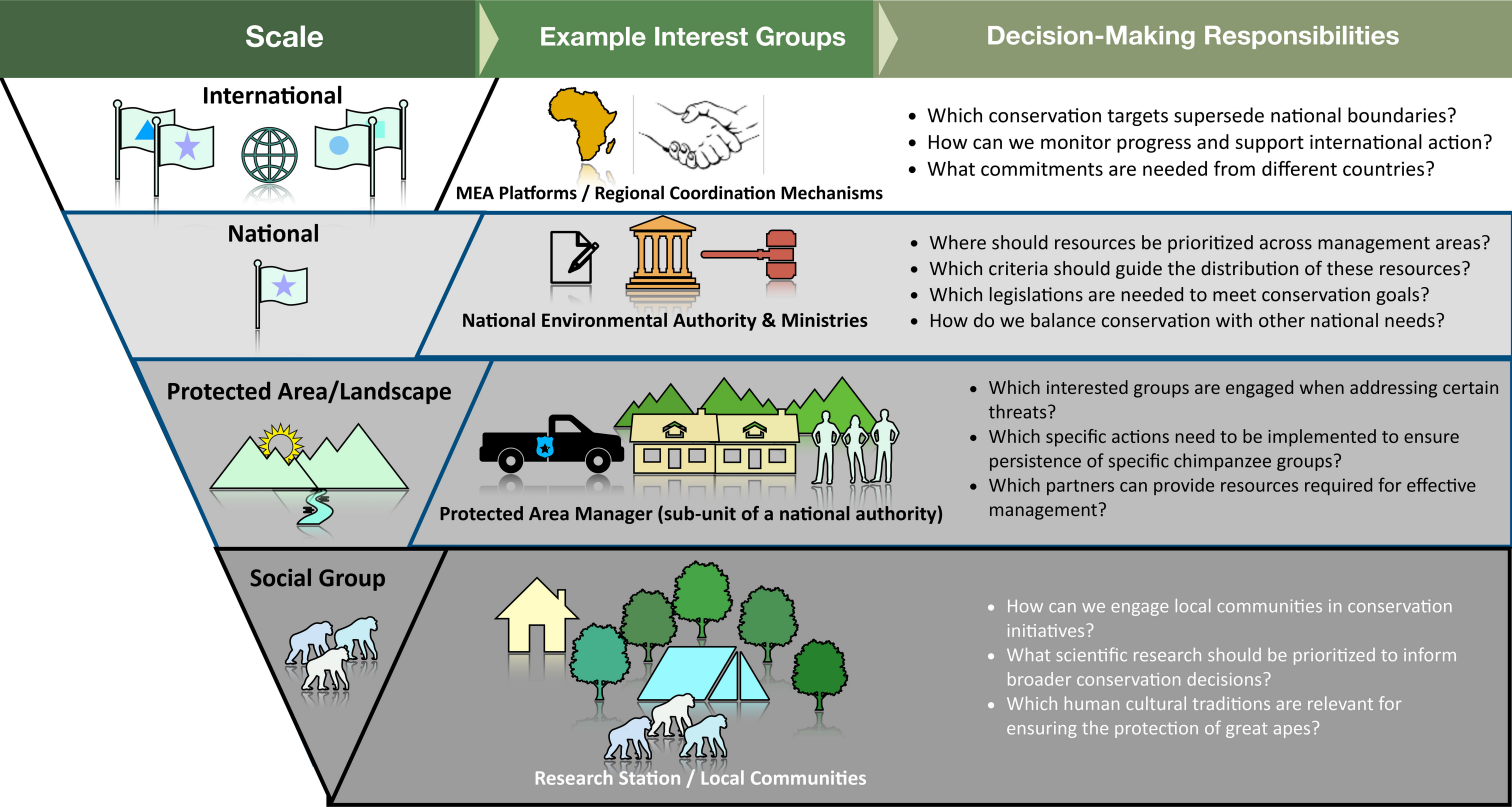
Scales of conservation decision-making and the potential relevance of animal culture to those scales, including examples of interested and affected groups who make these decisions.

There are significant efforts underway to counteract declines in chimpanzee populations, with conservation measures being implemented across multiple scales (e.g. national and regional conservation action plans) and sectors.^3^ Scale in conservation is of significant importance, as goals, targets, priorities and approaches are contingent on the scale under consideration [38]. At smaller scales, priorities are often defined and implemented for a local context, rarely considering how measures contribute to priorities of conservation for the taxon at larger scales (figure 2), while at larger, national or supra-national levels (e.g. [28]), conservation prioritization focuses on issues that are relevant to multiple management units (e.g. protected areas).

The scale of conservation action also determines the management organizations implicated. National-scale initiatives (e.g. national parks) are often overseen by a single management entity, whereas supra-national-scale initiatives (e.g. international wildlife corridors) hinge upon coordination across governments, international organizations and other affected groups. Examples of this are Multilateral Environmental Agreements (MEAs), like the UN Convention on the Conservation of Migratory Species of Wild Animals (CMS), where no single entity has authority over conservation prioritization or implementation, making inter-entity coordination of interested parties key for successful action. Further, with over one-third of the world’s remaining biodiversity occurring in indigenous territories and around local communities, and 65% of the world’s land being under indigenous or local community customary ownership and use [39], local governance authorities and community-based organizations are critical decision-makers for chimpanzee conservation^4^.

Chimpanzees have been highlighted as an effective umbrella and flagship species [42] and are the focus of many targeted conservation initiatives across management scales. For example, the International Finance Corporation (IFC) requires companies potentially impacting great apes in their project area to consult with the ARRC (avoid, reduce, restore and conserve) Task Force of the IUCN Species Survival Commission Primate Specialist Group’s Section on Great Apes (SGA; [43]). For chimpanzees, conservation strategies are often planned at the subspecies or regional level by the SGA and are commonly the focus of regional aid programmes (e.g. USAID’s West Africa Biodiversity and Climate Change programme focusing on western chimpanzees). Chimpanzees may also act as a priority taxon for the creation of new protected areas (e.g. Moyen Bafing National Park in Guinea). National-scale chimpanzee conservation strategies have been less consistently developed across chimpanzee range states and can take multiple forms (e.g. GRASP National Great Ape Survival Plans, National Biodiversity Strategies and Action Plans under the Convention on Biodiversity), creating a diverse patchwork of actions and prioritization schemes.

## 2. Diverse initiatives concerning chimpanzee culture and conservation

Behavioural flexibility allows chimpanzees to occupy a diverse array of habitats while also potentially promoting some resilience and adaptability to environmental change, including anthropogenic disturbance (e.g. [11]). Nonetheless, increasing anthropogenic pressure has been found to erode chimpanzee behavioural diversity [9], likely disrupting their capacity to cope with the manifold challenges they are facing (see above). Therefore, there is an urgent need to develop strategies to conserve not only chimpanzee populations but also their behavioural and cultural diversity.

As the extent of chimpanzee culture becomes increasingly apparent, and the conservation needs of the taxon more urgent, there is also a growing awareness that animal social learning and culture hold important implications for conservation planning, practice and policies [4–6,9,44]. Chimpanzees are one of the few species—and the only terrestrial one—for which the implications of culture have been formalized in domestic and international policy (alongside policies on several whale species (e.g. [45,46]). Therefore, chimpanzees offer a pioneering case study on the linkages between terrestrial animal culture and conservation, enabling evaluation of the transferability of approaches that have, until now, largely focused on marine taxa.

The knowledge accumulated over the past 60 years about chimpanzee behaviour and cultures, coupled with diverse conservation efforts across their range, provides a robust foundation for the productive integration of this knowledge into conservation practice. Conservation assessments have traditionally focused on population abundance and viability, along with habitat protection and landscape-scale spatial considerations, like connectivity. However, there is potential to incorporate cultural diversity (e.g. [47]), along with the social and environmental factors that enable social learning and transmission of behaviours, into a conservation framework and capitalize on all we have learned about chimpanzee culture to enhance awareness, funding and mobilize action for chimpanzee conservation.

How we choose to integrate culture into conservation will be scale- and goal-dependent. For example, we may use culture to improve prioritization decisions or other conservation policies at regional scales, or as a consideration in ecosystem management in protected areas. Ensuring that opportunities exist for chimpanzees to exhibit cultural behaviours could be a key factor when delineating units for conservation management [7]. Defining relevant thresholds for when a conservation unit, such as a taxon, population or culturally significant group, can be considered useful is critical for cultural conservation efforts. These thresholds include instances where cultural behaviours have clear and measurable effects on individual survival, reproduction or dispersal [5], where culture may hold wider evolutionary importance to the species [47], or where there is added value to indigenous people or local communities. The application of culture to different conservation scales is not inherently taxon-dependent (e.g. it may be easier to incorporate approaches for conserving animal culture for many species at local rather than taxonomic scales), but the forms these approaches take are likely to be especially specific to taxonomic context (e.g. in cetaceans the concept of what a ‘local’ approach may entail will be of a different scale from that for chimpanzees). For example, depending on the taxon, the definition of cultural units may be based on different lines of evidence. While in some cetaceans cultural units can be defined by a single stable cultural variant (e.g. a particular foraging strategy), the cultural diversity of chimpanzees and their conservation may necessitate a different approach that focuses on a whole suite of cultural behaviours.

Although the specific targets (i.e. the unit identified at which conservation efforts are directed to meet a conservation goal) for conserving chimpanzee cultures, and how culture can best serve wider conservation goals, are currently only in their earliest stages of exploration, defining and implementing these targets in such a well studied cultural species will likely prove valuable for similar efforts in other species [5]. In what follows, we describe key elements of these developments, including caveats and considerations for broader taxonomic application of the animal culture toolkit to conservation contexts. Given chimpanzees’ role as a charismatic flagship species, incorporating cultural considerations can have downstream impacts on conservation efforts at multiple scales beyond chimpanzee conservation alone [42]—but only if we are careful to ensure that these initiatives are constructed to be measurably effective in the long term [44].

### (a) A UNEP–CMS concerted action for the nut-cracking chimpanzee culture of West Africa

Advocacy for the importance of considering animal cultures in conservation policy was initiated by cetacean researchers, who proposed to supplement the traditional ecologically significant units with ‘culturally significant units’ ((CSUs) [47]). This concept was realized through the CMS, a UN treaty with over 130 state parties, designed to assist countries in collaborating in the conservation of species that migrate cyclically and predictably between range states. With regard to animal culture, the CMS began to explore how culture, social complexity and/or social learning may be important factors to consider in transboundary conservation efforts.^5^ While initial efforts focused on cetaceans (e.g. a Concerted Action (CA) on clans of sperm whales),^6^ the taxonomic scope was subsequently broadened to include terrestrial species. As a result, it was agreed to pursue another CA focused on the nut-cracking culture of western chimpanzees,^7^ given the multiple lines of convergent evidence that nut-cracking is socially transmitted [50], the behaviour’s suggested potential fitness benefits [51], and the richness of chimpanzee behavioural repertoires more generally [9,10,12].

Chimpanzees crack open a variety of species of nuts using natural stone or wooden hammer materials (figure 1). This provided an attractive target for exploring the conservation relevance of culture, and how best to coordinate cross-country management actions, given that the behaviour is exhibited by a single sub-species (i.e. *P. t. verus*) spanning an area across West Africa (i.e. Côte d’Ivoire, Guinea, Liberia and Sierra Leone, of which all but Sierra Leone are CMS parties). Nut-cracking behaviour has some of the strongest evidence supporting its cultural nature,^8^ making it a prime candidate for piloting applications in conservation efforts that focus on a specific cultural behaviour.^9^

Beyond the characteristics of the behaviour itself, an additional reason for focusing on nut-cracking was that the western subspecies (*P. t. verus*) is listed as ‘Critically Endangered’ [30]. Consequently, the nut-cracking traditions common in a subset of the western chimpanzee population may be even more vulnerable than other chimpanzee cultures. Further elements of the rationale were that chimpanzees and their nut-cracking technology represent a charismatic phenomenon to pilot culturally based conservation policy, with a potential to attract the attention of conservation policymakers and other interested groups,^10^ leverage funding and stimulate further developments in culture-linked conservation work with chimpanzees and other species. Certainly, such characteristics of a behavioural target are easily translatable to behaviours of other taxonomic groups.

Given these considerations, a proposal for ‘A Concerted action for the nut-cracking chimpanzees of West Africa’ was prepared by the CMS Expert Working Group and was approved at the CMS COP in Gandhinagar, India [52]. Parallel attention to the topic by various IUCN groups has subsequently led to new collaborations between CMS and IUCN parties and the approval of an expanded CA (described in §2c).

### (b) Approaches to chimpanzee culture and conservation under the auspices of the IUCN Section on Great Apes

Parallel to the efforts underway at CMS, the SGA began considering conservation targets beyond abundance (e.g. behavioural or genetic diversity) in their conservation strategies. This work included recognition of the potential role of culture in the long-term survival of great ape species. While previous conservation strategies have acknowledged that chimpanzees have cultural behaviours [53], the regional action plan for the western chimpanzee subspecies (Western Chimpanzee Action Plan (WCAP); [28]) represents the first formal integration of culture-specific initiatives in the SGA where cultural diversity was included as its own conservation target. The WCAP therefore defined related conservation activities, such as establishing a cultural diversity baseline for the taxon and increasing awareness about the value of cultural diversity preservation among interested or affected groups (e.g. local communities, protected area managers, governmental environmental authorities).

Since then, the SGA has continued to integrate cultural components into other aspects of conservation planning and policy. For example, in parallel to more traditional monitoring data, the creation of a behavioural diversity database via the A.P.E.S. Wiki [54] allows the storage of behavioural data collected during ape population surveys, providing a platform through which cultural diversity baselines can be facilitated. Regional planning workshops have subsequently prioritized the supplementation of behavioural data into monitoring schemes^11^ to further facilitate the creation of these baselines. Most prominently, however, a significant outcome of the SGA activities was the creation of the Working Group on Chimpanzee Cultures (WGCC) in 2021. This group, composed of experts in chimpanzee behaviour and conservation, guides and participates in efforts to incorporate cultural considerations into practice and policy for chimpanzee conservation.

Collectively, SGA activities, which largely focus on the long-term conservation of apes and seek to achieve the effective operationalization of culture into existing conservation frameworks, adopt a complementary approach to those of the CMS. There are some basic differences in their approaches, driven in part by differences in the taxonomic scope and scale of work of both groups. As an international platform, the CMS seeks to provide guidance to governments on how cultural behaviours relevant to conservation can be identified in any *migratory* species and integrated into inter-governmental conservation efforts, whereas the SGA uses *great apes* as a starting point from which to integrate culture into efforts, regardless of scale. Recent efforts by the IUCN and the CMS to collaborate on initiatives of overlapping interest have further strengthened development on this front. For example, SGA members integrated their independent activities into the outcomes of the CMS nut-cracking chimpanzee CA when planned efforts were stalled owing to the COVID-19 pandemic. This collaboration allowed its successful closure in conjunction with further collaboration on the development of a new CMS CA proposal entitled *‘*Concerted action for chimpanzee cultures and behavioural diversity’ to establish a formal collaborative framework to guide future efforts (see §2c; [54]). In addition, the WGCC has committed further efforts to define the pros and cons of various approaches to using culture for the conservation of ape species as an entry point for applications in other taxa (e.g. [7,44]), and subsequently outline the ways in which these approaches could be formalized and facilitated.

The CMS and IUCN have therefore proposed promising approaches for implementing cultural considerations into chimpanzee conservation efforts, and the factors necessary for these approaches to be effective. However, the success of these approaches will be judged based on their efficacy in achieving their intended outcomes. Thus, the intention of the CA for nut-cracking was to provide a test case for exploring the practicalities of improving conservation support to populations that exhibit well understood, culturally transmitted behaviours. More broadly, the CA was intended to bring increased political commitment to implement the actions already identified in the WCAP and hence to improve global conservation for chimpanzees and their cultures at large.

### (c) A concerted action for chimpanzee behavioural diversity and cultures

With the aim of effecting wider impact, the CMS Expert Working Group and the WGCC agreed to broaden their efforts in the conservation arena and propose a broader CA to supersede the previous one that focused exclusively on nut-cracking populations. The principal aim of this newer CA [55] is to define targets and develop effective approaches to conserve chimpanzee behavioural diversity and cultures across their range.^12^ Conserving biodiversity is a central pillar of conservation policies at large; correspondingly, the pervading role of culture in chimpanzee behaviour means that maintaining *cultural diversity* becomes an important supplementary target, in turn supporting the species’ overall capacity for adaptation and resilience in the face of anthropogenic and other environmental challenges. Considering the diverse cultural repertoires of chimpanzees, it is likely that many key indicators of cultural diversity span jurisdictional lines between CMS parties and therefore should be addressed through policies that transcend national and political boundaries. Conserving the ability of chimpanzees to express a diversity of behaviours, and/or sustaining current levels of behavioural diversity across the species, is designed to ensure that the benefits of initiatives inspired by the CA are broadly applicable to the species at large. This approach should help mitigate the prioritization dilemmas associated with behaviour-specific strategies (see §3a), by instead placing an emphasis on behavioural and cultural *diversity*.

With the collective expertise built by CMS and WGCC, and lessons learned from previous efforts, the new CA is designed to ensure that behavioural diversity and culture are effectively integrated within existing conservation and research frameworks with complementarity and long-term sustainability. The document outlines a pipeline through which behavioural and cultural diversity targets can be defined and integrated into practical contexts by a diverse range of parties [55]. While the CA is still in the process of implementation, its most challenging step is to determine appropriate targets for the conservation of chimpanzee behavioural diversity (see §3a). Once these targets have been defined, the document calls for a staged implementation of these targets, including a framework to foster the engagement of various interested groups in the preservation of cultural diversity. Proponents of the CA advocated that it is essential that initiatives growing from the CA are developed in concert with local community groups, practitioners and policymakers to ensure that they address active conservation needs, concerns and opportunities. Furthermore, the CA outlines plans to incorporate and specifically highlight traditional knowledge and understanding of chimpanzee cultures by indigenous and local people as a means to stimulate more diverse interest in and leadership of conservation initiatives. One way that the CA will facilitate this is by creating a collaborative network that will enable the co-creation of the frameworks necessary to understand, define and monitor behavioural diversity with practitioners and students based in chimpanzee range countries.

Ultimately, establishing a system to collect, aggregate and integrate findings into management schemes for the benefit of conservation targets is essential. This approach combines the process of data collection with practical considerations (e.g. prioritization, holistic conservation management) that can be applied at a range of scales, from on-the-ground action to inter-governmental policy. The proponents of the CA identified the A.P.E.S. Wiki as a clearinghouse that can aggregate information gathered across populations to identify patterns, shared threats and possibilities for cross-boundary conservation initiatives focused on culture [54]. The activities from the CA will establish practical standards (e.g. target definition, best practice guidelines, processes for the integration of data on cultures into decision-making pipelines) for integrating theoretical concepts into tangible guidelines that are useful to decision-makers [7]. Consultation with local practitioners tasked with effecting conservation decisions will be a core component of the development of these standards, ensuring that they remain grounded in practical management processes and directly address management needs.

Finally, coupled with the initiatives outlined in the WCAP [28], the CA’s planned activities align with the WCAP-mandated process of establishing cultural baselines to assess the efficacy of management approaches once they are implemented. By formalizing and standardizing conservation targets, defining processes to achieve these targets, and establishing a framework to measure their success, the 2024 CA represents a significant advance towards a clear and fair approach to integrating chimpanzee culture as a supplementary conservation target alongside standard conservation targets.

## 3. Recommendations to guide future strategies

In this section, we share insights gained from our collective experience with studying and conserving chimpanzee cultures that may help in guiding future efforts to incorporate cultural elements into conservation strategies for chimpanzees and other taxa. Rather than prescribe specific solutions for the complex decisions involved in the process of integrating culture into conservation (many of which are likely taxon- and context-specific), we offer a set of recommendations designed to assist in guiding the decision-making process in future initiatives, including considerations for the definition of conservation targets.

### (a) Precise definition of conservation targets

A conservation target can take many different forms when applied to animal cultures [7^7^], ranging from specific cultural variants (e.g. nut-cracking [52]) to species-wide behavioural diversity [9,55]. Effective pursuit of animal culture conservation targets is likely to require the preservation of the social and ecological conditions that allow the expression and maintenance of these behaviours. Thus, conservation actions may take the form of preserving the requisite social units, specific localities to which the variant is linked (e.g. termite mounds), or any materials necessary for the cultural behaviour to be expressed (e.g. a particular nut species for nut-cracking, or algae-producing pools for algae fishing). For example, to conserve nut-cracking cultures in chimpanzees, both the individuals that express the behaviour and the components of the tool task (e.g. hammer, anvil, nut) must be targets of preservation. In this sense, the definition of a conservation target as only the populations that perform a behaviour (e.g. nut-cracking chimpanzee populations: see [52]) would inherently encompass neither the behaviour itself nor the processes that would permit the behaviour’s retention. What is essential, therefore, is that conservation targets are based on clearly articulated conservation goals that have been formulated in collaboration with all interested and affected parties and tailored to the appropriate scale of implementation [44]. Examples of those goals could be to maximize taxonomic abundance, support processes that improve individual fitness or maximize the retention of taxonomic-wide cultural diversity. Each goal will necessitate a different suite of actions, including establishing how they complement other targets of conservation (e.g. abundance, genetic diversity; [9]).

### (b) Harmonization with traditional conservation strategies

Prioritization of targets across populations is an undeniably complex process and is a daily challenge faced by conservation managers. Resources for conservation are generally scarce relative to need [28,35,36]. Therefore, decision-makers at multiple scales must determine how to most effectively distribute limited resources, and, consequently, must prioritize some targets over others, guided by strategies at national, regional and other scales. Carvalho *et al*. [44] highlighted that any focus on cultural traits should complement traditional prioritization schemes, and policymakers must ensure that conservation of cultures does not serve as the only criterion for the allocation of scarce resources. In the ideal case, conservation support and funding would provide a multitude of both short- and long-term benefits to populations of cultural species if applied appropriately.

Although regional-scale landscape prioritization occurred in the past for western chimpanzees [56], proponents of the ongoing regional plan chose not to prioritize populations, instead leaving these decisions to national-level strategies [28]. However, very few countries in the region have developed national chimpanzee strategies (Guinea is a notable exception [57]). There is thus a risk that decision-makers might interpret advocacy for populations exhibiting a particular cultural behaviour (e.g. nut-cracking as through the initial CMS CA case study) as a call for assigning higher funding priority, especially where regional- and national-level prioritization strategies have not been formalized (e.g. Liberia, Côte d’Ivoire and Sierra Leone).

### (c) Modulating advocacy perspectives with objective data

Where national-level conservation and land-use planning strategies have been developed, international-level advocacy must be carefully dovetailed with those existing strategies. For example, in Guinea, nut-cracking is confirmed only for a small fraction of the chimpanzee range, such as the Bossou community of three remaining individuals (K. Koops, personal communication), and in nearby Diecke [58]. Considering chimpanzee distribution in Guinea [57], advocacy to enhance support specifically to nut-cracking populations would therefore contrast with conventional approaches to priority setting, especially in light of the regional distribution of the behaviour being prevalent beyond Guinea’s borders. Existing national schemes instead prioritize more northern areas of the country that collectively hold the largest populations of chimpanzees in West Africa [32,57], but these populations are not known to crack nuts (despite the local availability of consumed nut species; [59]). Accordingly, national policymakers must balance various advocacy perspectives with data that speak to other prioritization metrics to ensure resource distribution aligns with agreed conservation priorities at the national scale. Therefore, the rationale for conserving nut-cracking within Guinea’s borders presents a dilemma: should nut-cracking populations receive bespoke conservation owing to the intrinsic value of this behaviour, or should the complementarity of cultural targets to traditional conservation targets take priority? Clearly defining the goal for which ‘culture’ (whether a particular cultural behaviour or the capacity itself) can contribute as a conservation target will be critical in identifying the appropriate approach.

### (d) Overcoming knowledge inconsistencies and attention bias

Given that the challenges faced in dovetailing the conservation of behavioural entities with traditional metrics are not specific to nut-cracking,^13^ cultural preservation may most effectively motivate conservation where a population demonstrates a culture but is not otherwise considered high value by traditional metrics. However, integrating cultural traits into conservation schemes presents challenges around balancing advocacy and uneven knowledge, as we must know the behaviour of particular populations to advocate for them based on their culture [44]. Yet, commonly, the most highly valued populations already benefit from research attention, thus creating a feedback loop where advocacy may reinforce such biases. We currently have incomplete knowledge of cultural traits in less studied areas that could be overlooked without advocacy, even though those areas may harbour populations that stand to gain the most from incorporating culture into prioritization schemes. In an attempt to rectify these biases, it may be possible to extrapolate a behaviour’s presence across areas where we lack direct evidence, based on contextual information from the locations where the behaviour is known to occur.^14^ However, given that cultural behaviours are by definition driven by factors (e.g. social) that may not be easily captured by remotely sensed spatial data, such applications are likely limited.^15^

Without tools to rectify these biases, there is a need to bolster our understanding of chimpanzee cultural behaviours across their range, especially in areas lacking in conservation and research attention. Ultimately, it will be important that addressing biases and knowledge gaps does not detract limited resources away from direct conservation action [44,60], but can be integrated into existing data collection schemes, or can be interpolated from existing data. For example, providing the structure for behavioural monitoring to be integrated into traditional biomonitoring schemes offers a potential mechanism through which to fill research gaps, reduce attention biases and aid in the process of anchoring management strategies to more tangibly incorporate chimpanzee culture. At present, only about 28% of governmental environmental agencies in the range of the western chimpanzee subspecies regularly incorporate behavioural data in their management programmes (E. Wessling 2025, unpublished data), so there is room to integrate these approaches more formally into monitoring schemes. Incorporating behavioural monitoring can also serve as a platform for the empowerment of a range of local scholars and students by providing a framework for future conservationists to engage with chimpanzees as cultural beings. This approach not only facilitates the sustainable integration of culture in conservation work but also ensures the equitable inclusion of diverse voices in these discussions.

Additionally, long-term research sites may be the strongest opportunities to address the difficult challenge of documenting individual-level benefits of culture on fitness outcomes in a long-lived species like the chimpanzee [4,5]. Using the existing research platforms at those sites and centring long-term sites as responsible parties in contributing to conservation would strengthen the known positive effect of research monitoring stations as conservation beacons in a landscape [61].

Another approach to estimating the fitness benefits of culture is to adopt a population-level perspective commonly used in conservation monitoring. For example, estimating the impact of various cultural behaviours as predictors of conventional population-level success measures (such as population size, density, distribution, change and/or viability), alongside traditional environmental predictors (e.g. seasonality [10]) could be informative. This approach could also be applied to cultural or behavioural diversity, in a similar fashion to measurements of the impact of genetic diversity on such population metrics. For chimpanzees, several already available datasets (e.g. [9,12,17]), can facilitate such analytical approaches, potentially serving as valuable models for similar efforts in other species.

### (e) Establishing infrastructure to collate cultural data

Progress in recognizing and cataloguing behavioural diversity is likely more straightforward to evaluate than mapping the distribution of specific behaviours, particularly in cases where platforms like the A.P.E.S. Wiki are available to document cultural presence [54]. The A.P.E.S. Wiki is a platform for consolidating site-level information on ape status, threats, conservation efforts and ecological and behavioural data in an accessible manner [54]. This resource enriches existing databases (e.g. the A.P.E.S. database; [32]) by integrating additional site-specific records, such as observed behaviours, and consolidating data from individuals involved at each site, such as park managers and scientists. It also incorporates publicly available resources, like scientific and survey reports, centralizing the information essential for the challenging task of establishing cultural baselines from diverse reporting methodologies. Although the diverse origins and methodologies of contributions necessitate careful data harmonization, centralizing platforms like the A.P.E.S. Wiki streamline the creation of baselines by consolidating varied data sources, resulting in more efficient and meaningful outputs. Efforts to date have pushed the needle towards an established framework for collating existing information about chimpanzee cultures, and have helped create a pathway for how further data could be integrated into existing conservation schemes, as well as allow the framework from which baselines can be articulated and subsequently tied with conservation goals. Collectively, these initial forays provide valuable lessons to guide the next steps in these discussions.

### (f) Adopting more holistic approaches to chimpanzee conservation

Ensuring the long-term preservation of chimpanzees will require that the ecosystem networks, flora and fauna that make up the foundation of chimpanzee–environment interactions persist. Traditionally, regional environmental agencies have not prioritized as part of their conservation strategies protecting specific plant species that chimpanzees consume. However, advocating for the protection of a cultural behaviour, like nut-cracking, could encourage a more holistic ecological perspective, because it draws attention to the interconnected relationships between chimpanzees and their environments (here, with the nut-bearing tree species like *Coula*, *Sacoglottis* and *Panda* spp. that permit the behaviour to occur). While uncommon currently, advocating for a particular behaviour that depends on the presence of a specific food species provides a mechanism through which holistic ecological approaches that include not only the target species (i.e. chimpanzees) but also their food species (e.g. nuts or other dietary items) to population management can be prioritized. There are initial indications that such holistic management could feasibly be incorporated in management systems relevant to the western chimpanzee, as over half of environmental agencies (five of eight agencies) in West Africa actively manage arboreal species in their management approaches, most of which specifically cite tree species that are relevant to the chimpanzee diet (80%; E. Wessling 2024, unpublished data).

### (g) Raising awareness about animal cultures

Chimpanzee cultures have the potential to inspire greater interest and engagement in conservation among diverse groups. Certainly, while local communities and indigenous people likely have knowledge of chimpanzee behaviours like nut-cracking, they may not be aware of the uniqueness and importance of these behaviours to specific populations.^16^ Both CMS and SGA have previously advocated for wider dissemination of information on the values of cultural and behavioural diversity, as awareness-raising is one of the most easily funded and widely applied methodologies in western chimpanzee conservation [62]. Therefore, information dissemination about the value of cultural behaviours may be easily incorporated into and complementary to traditional educational approaches in the region (e.g. [63]), leveraging the charisma of the species. There is also good evidence from experimental settings that people demonstrate stronger support (e.g. larger donations, interest) for conservation measures of species that are familiar to them or generally charismatic [64,65]. Therefore, an increased focus on a range of charismatic behaviours when communicating with the public [44], such as the new approach adopted by the CMS and the SGA [55], should enhance our ability to garner effective species-wide support for chimpanzee conservation.

However, while awareness-raising may potentially garner support for conservation initiatives, there is as yet limited evidence of its long-term impact on effecting the necessary behavioural changes aimed at reducing the current threats to even the most charismatic species [66]. It remains difficult to evaluate how effectively such initiatives impact conservation decision-making or policy with measurable value to conservation outcomes. While no spatial prioritization exercises to date have incorporated cultural factors at public administrative scales for chimpanzees, the long lifespans of chimpanzees hamper our ability to assess if previous efforts on this front have reduced threats or improved the conservation status of chimpanzee populations. If awareness-raising continues, it will be imperative that campaigns are designed to pursue particular behaviour change outcomes, that they have carefully tailored messaging, and that they are executed skilfully with the most appropriate target audiences. Approaches to raising awareness about cultural behaviours will depend heavily on the audience—whether soliciting funding from the general public, incentivizing government officials to prioritize the conservation of particular species, or empowering civil society to advocate for the preservation of their local environment. Evaluating how best to harness the power of marketing the familiarity or charisma of cultural behaviours will determine how effective cultural behaviours will be in aiding us in addressing conservation targets.

## 4. Conclusions and future directions

Our overall aspiration is for the culture concept to be effectively integrated into conservation practice and policy. While the value of animal cultures is being increasingly recognized by conservationists, and some initiatives have been developed to preserve specific animal populations, demonstrating conservation gains remains challenging. This is at least partly due to the difficulty of defining conservation targets and the incomplete integration of the culture concept into scale-appropriate conservation frameworks. There has been progress in developing policies and mandates to incorporate behavioural monitoring in landscape-scale assessments, but much work remains to be done to make this information both useful and accessible. The joint IUCN–CMS initiatives on nut-cracking chimpanzee populations, and more recently, on chimpanzee cultural diversity more broadly, provide examples of how conservation practices can be extended beyond traditional metrics of population size and distribution. Building on lessons from these pioneering efforts, the approach of using culture as a mechanism for facilitating chimpanzee conservation across jurisdictional boundaries is now further conceptualized and ready for testing through the recently approved ‘Concerted action for chimpanzee cultures and behavioural diversity’ [55].

There is a clear urgency to conserve chimpanzee cultures both for their potential fitness benefits and for their intrinsic value to humans, including their relevance to local communities and indigenous people. Broader inclusion of local communities, conservation practitioners and other interested and affected groups is critical not only for ensuring the feasibility of incorporating culture in conservation but also for assuring its relevance in the local contexts where these animals live. Previous discussions about the definitions of animal cultures have largely been restricted to academic circles [44,67], with little translation to conservation applications (but see [5,47]). There have also been few initiatives that have explicitly involved indigenous groups or local communities as leading voices in the conservation of animal cultures. However, there are civil society organizations with a regional scope for great ape conservation that work directly with grassroots groups in community-led conservation initiatives for apes (e.g. Alliance for the Great Apes Conservation in Central Africa) that would be well suited to help shepherd initiatives across scales and interested parties.

For chimpanzees and other wildlife, spatial prioritization can occur at varied scales, from landscape, to national, to supra-national. Embedding culture into decisions shaping the delineation of management units (e.g. CSUs), or the prioritization between management units, could potentially aid conservation efficacy depending on specific goals, if implemented appropriately [7]. However, the efficacy of any approach will depend not only on how well the goals are defined but also on how implementable and accessible subsequent approaches are to practitioners and decision-makers across suitable scales. Beyond accessibility, the time scale of implementation will also be an important consideration given the ongoing pace of biodiversity losses [44]. Consequently, decisions made about how to approach the integration of culture into conservation must consider the speed at which data can be acquired to make robust, evidence-based decisions. As with many conservation projects, decision-makers will need to balance the inclusion of culture-forward approaches with a need to bypass decisions that necessitate slow data-collection processes. Naturally, conservation pipelines that heavily depend on further data collection should be minimized. Furthermore, communication with scale-appropriate organizations and groups will be critical to ensuring that actions are suitably implemented, as will be the explicit definition of measures of success, to guide and monitor progress towards these goals [44].

To address issues of scale, future efforts should adopt an integrated approach that considers the unique needs and decision-making processes at each level of governance. At local scales, initiatives may be best placed to focus on community engagement, behavioural monitoring, and facilitating the intersection of community livelihood support with animal cultures. Protected area managers could implement monitoring protocols to track cultural behaviours within their boundaries, guided by national-level standardized monitoring protocols and the integration of these data into national strategies and action plans. Internationally, organizations like CMS and IUCN can facilitate regional or trans-boundary coordination, data sharing between countries, and guidance on scale-appropriate indicators and targets. Adopting an integrated, multi-scale approach that meaningfully involves local communities can help operationalize cultural considerations into effective, sustainable conservation for chimpanzees in the future.

There are many potential benefits of stronger partnerships among range states to implement specific conservation activities to preserve chimpanzee behavioural and cultural diversity that have yet to be realized, but could have significant impacts on the effectiveness of conservation practice and policy. The complementary efforts of CMS and the IUCN provide a promising means of advancing cross-boundary policy initiatives with practical applications for chimpanzee conservation. As one of the few international bodies formally prioritizing the consideration of behaviour in conservation, CMS is well positioned to facilitate complex, collaborative engagement with this issue across a diverse network of groups and organizations. The SGA’s complementary efforts to formalize and update ape conservation action plans across ape ranges, to facilitate the WGCC, and to create an Animal Cultures Task Force will ensure responsible implementation of these initiatives. While the aims and policies are in place to conduct cross-boundary conservation of chimpanzee cultures, the implementation of these plans requires significant and sustainable sources of financial support.

There is considerable potential to leverage the public’s interest in animal cultures to garner support for conservation initiatives. Many people may recognize the inherent value of chimpanzees as cultural beings who share a recognition for aspects of their own behaviour. Others may be intrigued by behavioural differences among groups and across species. Regardless of the motivation, public interest should be leveraged in raising awareness and support for the long-term preservation of animal cultures.

## Data Availability

Supplementary material is available online [68].

## Footnotes

^1^
While not wholly explained by ecology, specific cultural repertoires of chimpanzees are often associated with local ecological opportunities [16].

^2^
The *IUCN Red List* is the authoritative guide for assessing species’ threat levels and conservation statuses globally, relying on IUCN-affiliated experts to evaluate criteria such as population decline rates, geographical range size and fragmentation and population size and trends. For example, the *Red List* assessment of ape species is performed by the IUCN Species Survival Commission Primate Specialist Group’s Section on Great Apes (SGA). The accuracy of *Red List* assessments depends heavily on the availability of sufficient data to determine if population trends justify a specific *Red List* categorization. Although western chimpanzees are estimated to have higher overall numbers than other ape taxa, they were uplisted from ‘Endangered’ to ‘Critically Endangered’ in 2016 [30], based on robust evidence of a significant population decline [31].

^3^
Many efforts are also underway at all levels outside of what would traditionally be considered conservation to address the drivers of threats to chimpanzee habitats (e.g. transforming cocoa supply chains).

^4^
For example, in western Tanzania, village land-use planning resulted in the designation of Village Land Forest Reserves and District Local Authority Forest Reserves, contributing to an increase in protection from 9% in 1994 to 43% of the chimpanzee range in Tanzania by 2019 [40,41].

^5^
Recognizing the potential implications of scientific discoveries about animal cultures, a resolution was passed at the 2014 ‘Conference of the Parties’ (COP) in Quito, Ecuador, requesting the CMS Scientific Council to establish an intersessional Expert Working Group that would address the conservation implications of animal culture and social complexity with an initial focus on cetaceans.

^6^
One mechanism to explore specific issues is the proposal of a ‘Concerted Action’ (CA) for adoption at the international CMS COP. The 2017 COP in Manila approved a landmark CA focused on acoustic clans of sperm whales. This decision represented the first recognition and exploration within a multilateral environmental agreement of the prospect that a population unit might be defined by cultural, rather than genetic, distinctiveness. At the same time, the taxonomic scope of work was broadened to cover all taxa listed on the CMS appendices [48].

^7^
While chimpanzees and other primates are not migratory in the biological sense, gorillas and chimpanzees are among the species added to the CMS appendices because they meet the legal definition of ‘migratory’ under the Convention [49].

^8^
The distribution of nut-cracking is not explicable through ecological factors alone, as appropriate nut species occur beyond the range of the behaviour [16]. Similarly, genetic explanations for these population differences in the existence of nut-cracking are discounted through behavioural experiments with *P. t. schweinfurthii*, which do not crack nuts in the wild, yet learn the skill once they observe another chimpanzee nut-cracking [50].

^9^
With respect to ‘intrinsic value’ [6,7], the chimpanzee nut-cracking CA appealed to a comparison with initiatives to conserve diminishing and endangered human languages. With respect to implications for vital rates (fitness), the CA cited research reporting that nut-cracking aids marginal populations in surviving seasonal periods of fruit scarcity [51].

^10^
Representatives of the CMS parties concerned have also publicly endorsed the implementation of the approved CA.

^11^
Similarly, the SGA convened a workshop in 2022 to develop a formalized monitoring scheme for western chimpanzees where the practicalities of behavioural monitoring were also considered. To address this need, clear protocols for (cultural) behaviour monitoring and more targeted behavioural surveying in data-deficient landscapes were identified as specific objectives that will allow more direct integration of behavioural diversity into chimpanzee conservation.

^12^
This includes 21 African nations, 19 of which are CMS parties.

^13^
Algae fishing, for example, a behaviour that is locally restricted to areas of northern Guinea, may instead offer better congruence than nut-cracking with conventional conservation metrics; its distribution already overlaps with areas of high conservation value [32,57], while also conforming to selection criteria for suitable candidate pilot cultural behaviours (§2a). Therefore, conserving algae fishing might add little value to traditional conservation metrics.

^14^
Such approaches are common in species distribution modelling where occupancy must be estimated from few samples of known presence, and may necessitate an extensive understanding of the contexts that elicit the behaviour. Similarly to species distributional models, it may be possible to map the spatial distribution of specific behaviours and combine these data with ecological data from those locations, thereby allowing extrapolations of the likelihood of the occurrence of certain cultural patterns over the chimpanzee range based on ecological data alone (e.g. [11]). By overlaying information on the threats to chimpanzees across their range, it should then be possible to develop strategies to conserve conservation units representing chimpanzee cultural diversity.

^15^
In the case of nut-cracking, observations of this behaviour are relatively spatially clustered, so enhancing resources to surrounding landscapes could theoretically provide support to unknown nut-cracking populations. While regionally clustered, nut-cracking behaviour also appears to be relatively localized. For instance, while the small group at Bossou cracks nuts, the nearby and heavily threatened Nimba Mountains population of chimpanzees in Guinea does not (e.g. [16,18]). It is therefore unlikely to be feasible at this stage to efficiently generalize support for behavioural entities like nut-cracking in the absence of observations confirming their presence. Extrapolation may be a permissible method of reducing bias if inefficiencies in further data collection exceed potential inefficiencies of false positives (i.e. areas where environmental context should allow the behaviour to be exhibited, even if chimpanzees may not exhibit the behaviour).

^16^
Discussions of chimpanzee cultures during the SGA’s 2022 workshop (see §2b and note 11) indicate promising interest among regional resource managers in recognizing the inherent value of chimpanzee behaviours.
